# Enhanced suppression of *Stenotrophomonas maltophilia* by a three-phage cocktail: genomic insights and kinetic profiling

**DOI:** 10.1128/aac.01162-24

**Published:** 2025-01-22

**Authors:** Alisha N. Monsibais, Olivia Tea, Pooja Ghatbale, Sage J. B. Dunham, Mirjam Zünd, Jennifer Phan, Karen Lam, McKenna Paulson, Natalie Tran, Diana S. Suder, Alisha N. Blanc, Cyril Samillano, Joy Suh, Hanna Atif, Ethan Vien, Ryan Nguyen, Allene Vo, Shane Gonen, David Pride, Katrine Whiteson

**Affiliations:** 1 Department of Molecular Biology and Biochemistry, University of California Irvine 8788 , Irvine, California, USA; 2 Department of Pathology, University of California San Diego 8784 , La Jolla, California, USA; 3 Department of Medicine, University of California San Diego 8784 , La Jolla, California, USA; Johns Hopkins University School of Medicine, Baltimore, Maryland, USA

**Keywords:** bacteriophages, genomics, antimicrobial resistance, *Stenotrophomonas maltophilia*

## Abstract

*Stenotrophomonas maltophilia* is an understudied, gram-negative, aerobic bacterium that is widespread in the environment and increasingly a cause of opportunistic infections. Treating *S. maltophilia* remains difficult, leading to an increase in disease severity and higher hospitalization rates in people with cystic fibrosis, cancer, and other immunocompromised health conditions. The lack of effective antibiotics has led to renewed interest in phage therapy; however, there remains a great need for well-characterized phages, especially against *S. maltophilia*. In response to an oncology patient with a sepsis infection, we collected 18 phages from Southern California wastewater influent that exhibit different plaque morphology against *S. maltophilia* host strain B28B. We hypothesized that, when combined into a cocktail, genetically diverse phages would give rise to distinct lytic infection kinetics that would enhance bacterial killing when compared to the individual phages alone. We identified three genetically distinct clusters of phages, and a representative from each group was further investigated and screened for potential therapeutic use. The results demonstrated that the three-phage cocktail significantly suppressed bacterial growth compared with individual phages when observed for 48 h. We also assessed the lytic impacts of our three-phage cocktail against a collection of 46 *S*. *maltophilia* strains to determine if a multi-phage cocktail has an expanded host range. Our phages remained strain-specific and infected >50% of tested strains. In six clinically relevant *S. maltophilia* strains, the multi-phage cocktail has enhanced suppression of bacterial growth. These findings suggest that specialized phage cocktails may be an effective avenue of treatment for recalcitrant *S. maltophilia* infections resistant to current antibiotics.

## INTRODUCTION

Antimicrobial resistance (AMR) in a clinical setting occurs when infecting microbes overcome antimicrobial medication, ultimately leading to severe disease and mortality in the infected patient. By 2050, AMR is projected to contribute to over 10 million deaths annually, leading to an economic impact of $300 billion as treatment will be prolonged and less effective (1, 2). This impending crisis has been connected to the misuse and overuse of antibiotics in the clinical setting and agriculture industry (3). Reduced investment in antibiotic discovery has also intensified AMR infection rates and impacts (4, 5). Phage therapy is a promising approach with the potential to mitigate these hard-to-treat recalcitrant infections (6).

Phage therapy utilizes lytic bacteriophages, viruses that infect bacteria, to reduce bacterial burdens associated with infections (7). Phages attach to the host bacterial cell via specific receptors, inject phage DNA, and hijack host machinery, ultimately resulting in host cell death by lysis and progeny virus release (8, 9). Although phages have been studied and used to treat bacterial infections for more than a century, basic research into phage safety, antibacterial properties, and best practices for therapeutic use have been understudied (10, 11). However, with the rise in AMR infections and the increased use of therapeutic phages, basic phage biology has taken on new importance. A special interest lies in understanding how phages successfully combat multidrug-resistant (MDR), extensively drug-resistant (XDR), or pan-drug-resistant (PDR) bacteria. MDR bacteria are classified as having resistance to at least one antibiotic agent in three or more categories, whereas XDR bacteria are resistant to all but one or two antimicrobial categories, and PDR bacteria are resistant to all antibiotic agents (12). Indeed, phage therapy has shown promising results in life-threatening infections in various MDR bacteria (6, 13, 14), and several clinical trials are currently underway (15, 16).

The development of safe and effective phages for therapy will benefit from the thorough characterization of phage infection kinetics, which are time-dependent interactions that reveal the phage replication strategy. Phage infection kinetics can be understood via one-step growth curves, which track phage adsorption rate and propagation (17, 18). These measurements reveal vital infection parameters, including the length of the latent phase, burst size, and duration of phage infection. These time-dependent phage-bacteria interactions are essential for identifying underlying phage selection pressures and antibacterial properties, which may strengthen phage therapy.

Current data suggests that individual phages generally have a narrow host range, meaning they can only infect a subset of strains from a single bacterial species (7, 19). Bacteria resist phage through several mechanisms, including restriction-modification systems, CRISPR-Cas9 immunity, abortive infection, and phage receptor modification (20–22). Thus, when host bacteria are exposed to single phages, previous data have shown resistance can quickly arise. An alternative strategy to mitigate this bacterial resistance is using a multi-phage cocktail. Multi-phage cocktails enhance the lytic outcomes with MDR bacteria since bacteria struggle to evolve resistance to several phages concurrently (23–26). Indeed, prior work in our and other laboratories has demonstrated that cocktails increase phage infectivity by reducing the growth of the target pathogen and limiting the development of phage resistance (24, 27, 28). Thus, designing cocktails is an essential aspect of improving the efficiency of phage therapy.

*Stenotrophomonas maltophilia* (*S. maltophilia*) is a gram-negative emerging opportunistic pathogen that has negatively impacted immunocompromised individuals and people with cystic fibrosis (CF) (29). *S. maltophilia* is innately antibiotic-resistant, containing an extensive repository of AMR mechanisms such as biofilm formation, beta-lactamases, and multidrug efflux pumps (30–32). Additionally, clinical isolates have higher mutation rates than their environmental counterparts, enabling them to adapt quickly (33). A recent meta-analysis of *S. maltophilia* global prevalence revealed an increased trend of *S. maltophilia* infections over the last 30 years, along with increased antibiotic resistance to both tigecycline and ticarcillin-clavulanic acid (34). Thus, there is a clear need to investigate phages against *S. maltophilia*. As of this writing, around ~120 phages have been reported to the National Center for Biotechnology Information (NCBI) with complete genomes (accessed 11 December 2024), and phage cocktails are understudied (35–46).

We hunted for phages in Southern California sewage influent and found 18 phages that could infect an *S. maltophilia* strain isolated from an oncology patient’s blood. We used these phages to address the following questions: (1) How genetically diverse are these 18 phages? (2) What are the phage infection kinetics of genetically distinct *S. maltophilia* phages? and (3) Can a phage cocktail comprising several genetically distinct phages expand lytic activity and effectively suppress bacterial growth? We hypothesized that genetically diverse phages would give rise to distinct infection kinetics, with differing adsorption time, latent phase, and lysis timing. We also explored whether a cocktail composed of phages with distinct infection kinetics can enhance lytic capacity compared with individual phages alone.

## RESULTS

### Comparative genomic analysis and bioinformatic screening of *S. maltophilia* phages

**Fig 1:**
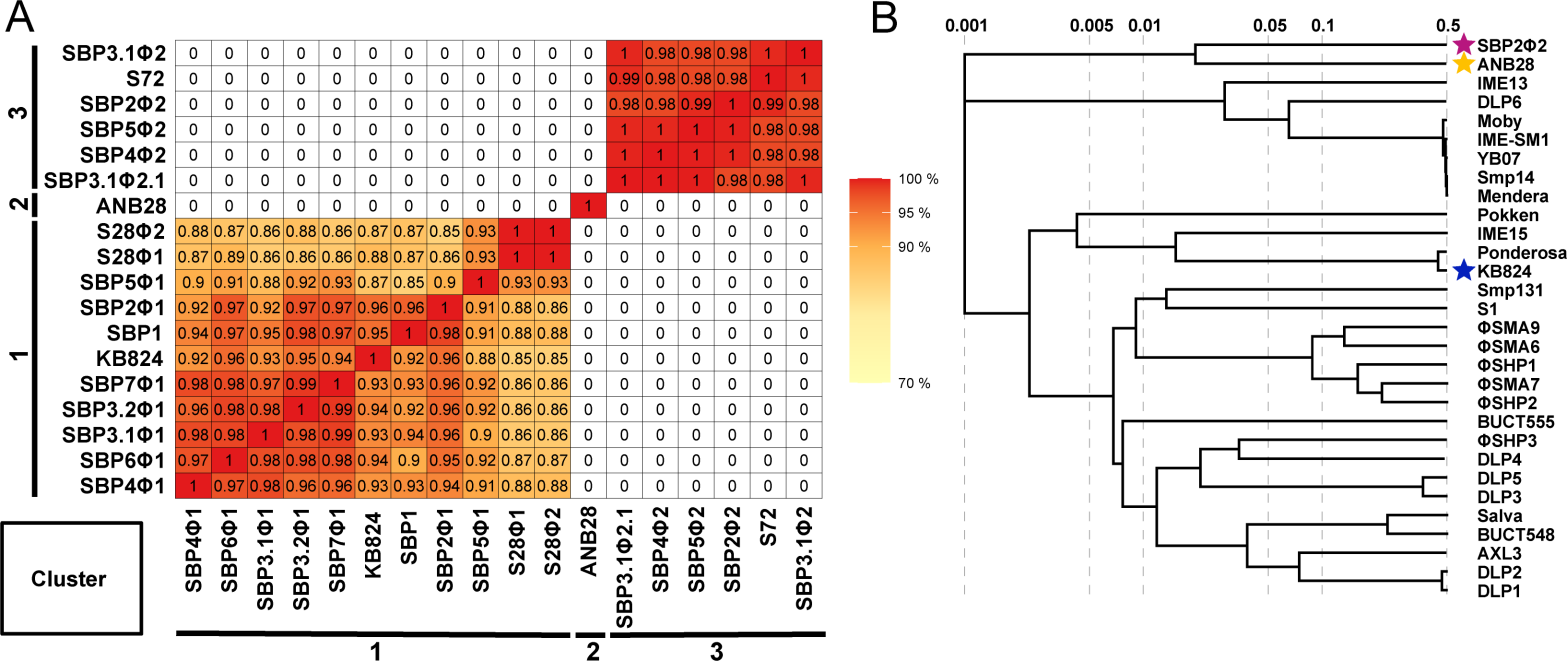
Comparative genomic analysis of *S. maltophilia* phages. (**A**) ANI% of our 18 isolated *S. maltophilia* phages. Phage sequences were cleaned and deduplicated with bbtools, assembled with unicycler, and annotated with RASTtk to obtain GenBank files (50–52). GenBank files were then processed with ANVIO using the “anvi-compute-genome-similarity” option to determine the ANI% (53). Cluster 1 (bottom left) represents podoviruses, ANB28 (Cluster 2) is a singleton phage, and Cluster 3 (top right) represents siphoviruses. (**B**) Phylogenetic analysis of our three *S*. *maltophilia* phages against known *S. maltophilia* phages previously isolated from the literature using VIPtree (35, 36). (Magenta star indicates SBP2Φ2; yellow indicates ANB28; and blue indicates KB824.)

We isolated 18 phages from Southern California wastewater influent against *S. maltophilia* strain B28B, a bacterial isolate from an oncology patient (Tables S1 and S2). Coverage analysis was conducted on each phage to ensure adequate coverage of sequencing reads (Fig. S1), and CheckV analysis was used to determine the completeness of the genome (Table S3). The average nucleotide identity percentage (ANI%) of the 18 phages in our *S. maltophilia* phage collection revealed three distinct phage clusters, one of which was a singleton isolate (Fig. 1A), which was also confirmed with the Virus Intergenomic Distance Calculator (VIRIDIC) (Fig. S2). Additionally, Basic Local Alignment Search Tool for nucleotide (BLASTn) analysis was conducted on each phage to assess similarity to previously identified phages (Table S4). The top right cluster, labeled Cluster 3, contained a high degree of similarity (>98% ANI%), and a siphovirus morphology was indicated by collective BLASTn hits to Caudoviricetes sp. isolate 94, Caudoviricetes sp. isolate 231, Caudoviricetes sp. isolate 163, *Stenotrophomonas* phage CUB19 (46), and Siphoviridae environmental samples clone NHS-Seq1. The bottom left phage cluster, denoted Cluster 1, contained variation in similarity with 86–99 ANI%, and a podovirus morphology was indicated by collective BLASTn hits to *Stenotrophomonas* phage Ponderosa (47), *Stenotrophomonas* phage Ptah (48), *Stenotrophomonas* phage Pepon (49), and *Stenotrophomonas* phage TS-10. Phage ANB28 was a stand-alone phage isolate labeled Cluster 2; BLASTn analysis of a 10% query coverage shared a 73.9% nucleotide identity to *Xanthomonas* phage JGB6, although the phage morphology was unknown. A comparative genomic analysis of each cluster was conducted with the top BLASTn hits (Fig. S3). Cluster 1 (proviruses) showed high similarities and coverage with previously identified phages (Fig. S3A). In contrast, the top BLASTn hits to the phages of Cluster 3 (siphovirus) had sparse coverage, suggesting novelty (Fig. S3C). ANB28 comparative genomics demonstrated an overlap in gene clusters with *Xanthomonas* phage JGB6 and a distinct ANI% (Fig. S3B). These results highlight that our *S. maltophilia* phage collection contains three genetically distinct clusters, including two novel phages.

**Fig 2:**
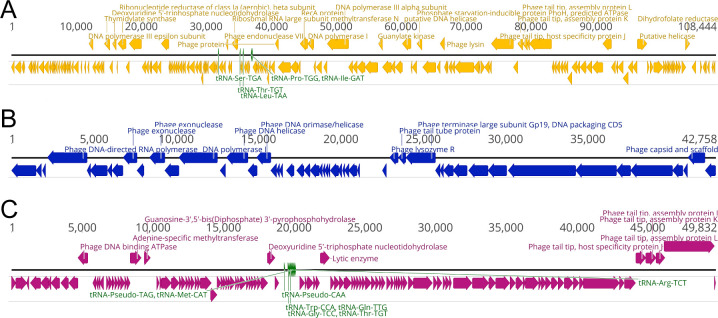
Genomic map of our three distinct phages. (**A**) ANB28 (yellow), (**B**) KB824 (blue), and (**C**) SBP2Φ2 (magenta). GenBank files were visualized in Geneious to establish phage genome maps (57). The annotated open reading frames (ORFs) are indicated with arrows above or on the black genomic line, whereas unlabeled hypothetical proteins are shown below. tRNAs are highlighted in green text.

After comparative genomic analysis, we selected one representative from each group: ANB28, SBP2Φ2 (siphovirus), and KB824 (podovirus). Phylogenetic analysis was performed using our three phages against 27 previously discovered *S. maltophilia* phages using ViPtree, a program to generate viral proteomic trees based on genome-wide similarities derived from tBLASTx (54). ANB28 and SBP2Φ2 diverge from previously isolated *S. maltophilia* phages, whereas KB824 is closely related to *Stenotrophomonas* phage Ponderosa, consistent with BLASTn results (Fig. 1B). Bioinformatic safety screening of the genomes from the three representative phages revealed no genome-encoded integrase, AMR, or toxin genes (Table 1). ANB28 had the largest genome at 108 kb, which consisted of 194 open reading frames (ORFs) and five tRNAs. KB824 had the shortest genome at ~43 kb, which consisted of 76 ORFs and zero tRNAs. SBP2Φ2 had a genome of ~50 kb, which consisted of 123 ORFs and eight tRNAs. Annotations of gene maps for each phage were created by listing genes with predicted annotations on the top row, unlabeled hypothetical proteins on the bottom row, and tRNAs denoted in green located on the genome line (Fig. 2). These phylogenetic and genomic results confirmed that our three selected phages are genetically distinct from one another.

**TABLE 1:** Summary of genomic information*^a^*^,^*^b^*

	ANB28	SBP2ɸ2	KB824
Length	108,444	49,832	42,758
GC%	53.3	52.1	59.8
ORFs	172	112	52
tRNAs	5	8	0
HP	148	103	41
Integrase genes	0	0	0
AMR genes	0	0	0
Toxin genes	0	0	0

^*a*^ After DNA extraction and assembly, genomes were analyzed for bacterial AMR genes using the Comprehensive Antibiotic Resistance Database (CARD) (55) and bacterial toxin genes using TAfinder (56). RASTtk annotations were performed and assessed in Geneious for integrase genes, hypothetical proteins (HP), transfer RNAs (tRNAs), open reading frames (ORFs), GC%, and length (52, 57).

^*b*^ HP, hypothetical protein; ORF, open reading frame.

### Basic morphological characterization of three distinct *S. maltophilia* phages

**Fig 3:**
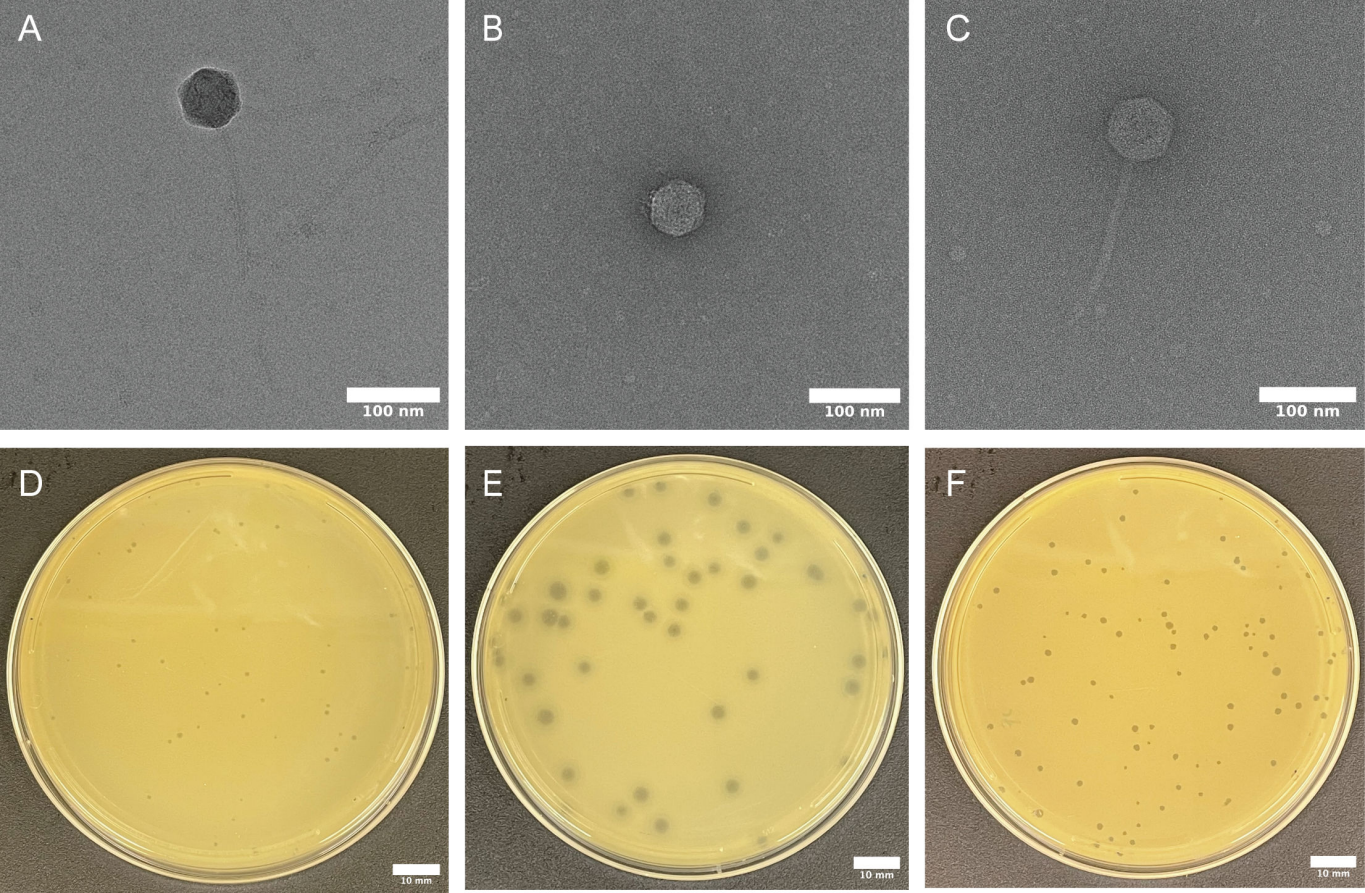
Phage morphology. (**A–C**) EM micrographs of negatively-stained high-titer phage lysate (>10^8^ PFU/mL) samples (58). (**D–F**) Phage plaque morphology on brain heart infusion (BHI) soft agar overlay with *S. maltophilia* bacterial lawns. Log phase bacteria were mixed with phages at a dilution to achieve countable plaque-forming units (PFU) and incubated at 37°C for 18–20 h. Scale bars represent 10 mm. (**A and D**) ANB28 has siphovirus morphology and pinpoint plaques. (**B and E**) KB824 has podovirus morphology and hazy-halo plaques. (**C and F**) SBP2Φ2 has siphovirus morphology and pleomorphic plaques.

Electron microscopy (EM) micrographs illustrate ANB28 as having a siphovirus morphology. KB284 and SBP2Φ2, initially classified based on sequence similarities, were confirmed by EM as having podovirus and siphovirus morphology, respectively. All three phages showed an icosahedral capsid, whereas both siphoviruses, ANB28 and SBP2Φ2, contained long, non-contractile tails. KB824, a podovirus, contained a very short non-contractile tail (Fig. 3A through C). Plaque morphology heavily depends on culture conditions, incubation times, and temperature. In our study, plaque morphology was only evaluated using Brain Heart Infusion (BHI) petri plates with a 0.35% low melting point soft agar overlay, incubated at 37°C for 18–20 h before imaging, except for phage KB824, which was also evaluated at room temperature. Plaque morphology for each phage was distinct: ANB28 makes pinpoint plaques, KB824 consists of hazy mid-size plaques, and SBP2Φ2 plaques are clear and pleomorphic (Fig. 3D through F). KB824 exhibited robust lytic activity at room temperature, showing variation in plaque morphology compared to 37°C (Fig. S4).

Efficiency of plating (EOP) analysis was conducted against 13 clinically relevant *S. maltophilia* strains to assess the host range of each phage using soft agar overlays. A high titer of ANB28 (>10^6^ plaque-forming units per milliliter (PFU/mL)) infected three *S*. *maltophilia* strains, including the *S. maltophilia*-type strain, K279a. KB824 had the broadest host range, with five *S*. *maltophilia* strains susceptible to a 10^5^ PFU/mL titer. SBP2Φ2 had the narrowest host range, consisting of only two *S*. *maltophilia* strains at a 10^5^ PFU/mL titer (Table 2). These results indicate that the three newly discovered phages could infect six of the 13 strains tested on solid media, with each exhibiting a different plaque morphology.

**TABLE 2:** Efficiency of plating (EOP) for *S. maltophilia* phages on *S. maltophilia* clinical strains^*a*^

Phage	PFU/mL	B28B	B28S	K279a	SM12LS	SM49LS	SM50JS	Others
ANB28	10^3^	**-**	**-**	**-**	**-**	**-**	**-**	**-**
ANB28	10^4^	**+**	**+**	**-**	**-**	**-**	**-**	**-**
ANB28	10^5^	**+++**	**+++**	**-**	**-**	**-**	**-**	**-**
ANB28	10^6^	**++++**	**++++**	**+**	**-**	**-**	**-**	**-**
ANB28	10^7^	**++++**	**++++**	**++**	**-**	**-**	**-**	**-**
KB824	10^3^	**-**	**+**	**-**	**+**	**+**	**-**	**-**
KB824	10^4^	**+**	**++**	**-**	**++**	**++**	**-**	**-**
KB824	10^5^	**+++**	**+++**	**-**	**++**	**+++**	**+**	**-**
KB824	10^6^	**++++**	**++++**	**-**	**+++**	**++++**	**+**	**-**
KB824	10^7^	**++++**	**++++**	**-**	**+++**	**++++**	**++**	**-**
SBP2Φ2	10^3^	**-**	**+**	**-**	**-**	**-**	**-**	**-**
SBP2Φ2	10^4^	**-**	**++**	**-**	**-**	**-**	**-**	**-**
SBP2Φ2	10^5^	**+**	**++++**	**-**	**-**	**-**	**-**	**-**
SBP2Φ2	10^6^	**++**	**++++**	**-**	**-**	**-**	**-**	**-**
SBP2Φ2	10^7^	**++++**	**++++**	**-**	**-**	**-**	**-**	**-**

^*a*^ -, no sensitivity to phage; +, few individual plaques; ++, turbidity throughout the cleared zone; +++, lysis with few resistant bacterial colonies; ++++, complete lysis of bacterial lawn. *S. maltophilia* strains: B28B, B28S, K279a, SM12LS, SM49LS, SM50JS. Other: SM15KA, SM17LS, SM20TB, SM22TB, SM26KA, SM27KA, and SM71PII.

### Phage infection kinetics of the three distinct *S. maltophilia* phages

**Fig 4:**
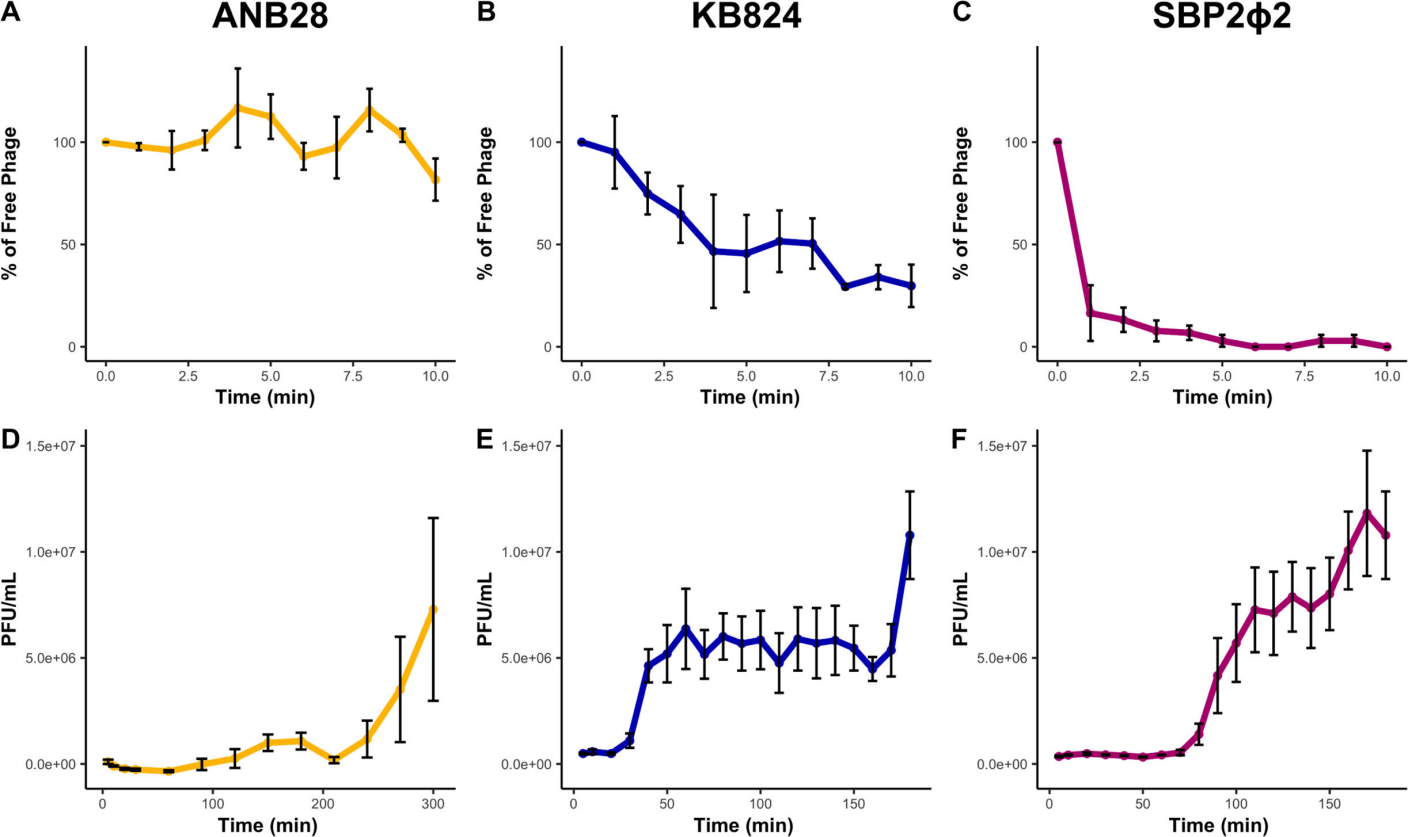
Phage kinetics of *S. maltophilia* phages against host strain B28B. (**A and D**) ANB28, (**B and E**) KB824, and (**C and F**) SBP2Φ2. (**A–C**) The rate of phage adsorption to bacterial host cells was measured using the rate of attachment protocol (17). Sampling was conducted every minute over 10 min using log phase bacteria (OD600 0.3) exposed to phage at MOI 0.001 at 37°C, shaking. The percentage of free phage was calculated by dividing the raw data at each time point by the average of the control samples lacking bacteria, multiplied by 100. (**D–F**) One-step growth curves were conducted for 3 or 5 h at 37°C, shaking; the samples were taken every 10–30 min (18). Log phase bacteria (OD600 0.3) were exposed to phage at MOI 0.001, and sampling was quickly followed by plating with a soft agar overlay. All plates were incubated at 37°C for 18–20 h before counting plaques. Three biological replicates were averaged and graphed; error bars represent standard error.

Phage kinetic assays, including rate of attachment and one-step growth curves, were conducted for each of the three *S*. *maltophilia* phages on host bacteria B28B at physiologically relevant body temperatures (~37°C) using a multiplicity of infection (MOI) of 0.001. The results indicated that each phage exhibits distinct adsorption rates to the host cell: SBP2Φ2 rapidly adsorbs within 5 min, and KB824 requires over 10 min for effective adsorption. ANB28 displays inefficient adsorption with only a subfraction of virions adsorbing even after 10 min. KB824 and SBP2Φ2 both followed first-order kinetics, whereas ANB28 showed a slower adsorption process (Fig. 4A through C). For the one-step growth curves, ANB28 had the most prolonged latent period of around ~90 min, with an average absolute burst size of ~1 × 10^6^ PFU/mL for the initial burst. Interestingly, ANB28 returned to a latent phase immediately after the initial burst, followed by a larger burst of progeny virus from the host cell, demonstrating a variable multi-cycle curve. KB824 had the shortest latent period, ~30 min, with an average absolute burst size of ~5 × 10^6^ PFUs/mL. SBP2Φ2 had a latent period of ~80 min with the largest absolute burst size of ~7 × 10^6^ PFUs/mL (Fig. 4D through F). The results indicate that each lytic phage has distinct infection kinetics, such as the adsorption rate, latent period, and absolute burst size.

**Fig 5:**
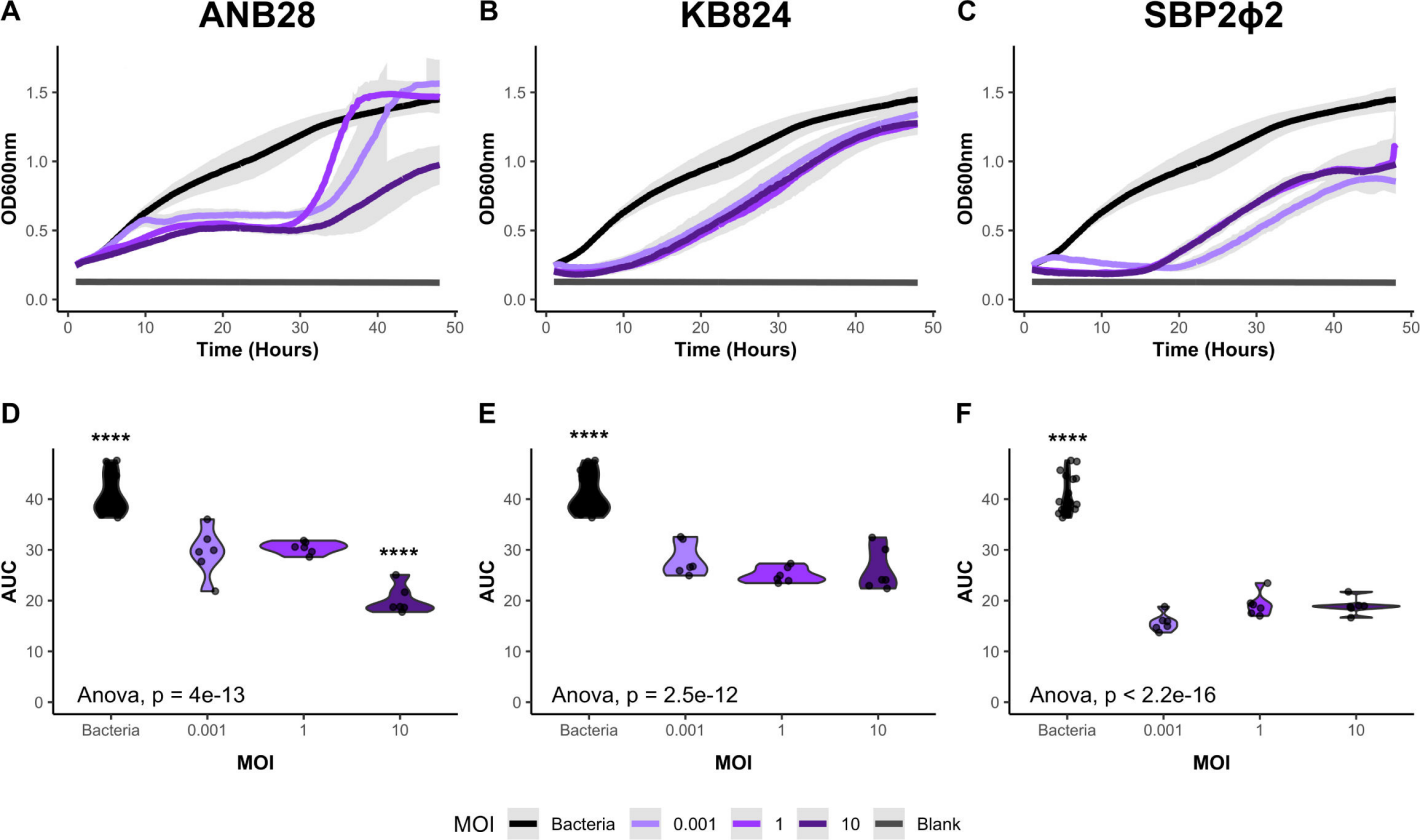
Impacts of varying multiplicity of infection (MOI) against *S. maltophilia* host strain B28B. (**A and D**) ANB28, (**B and E**) KB824, and (**C and F**) SBP2Φ2. (**A–C**) Growth curve analysis of bacteria B28B in the presence of three isolated phages. Log phase bacteria (OD600 0.1) were added to 96-well plates and exposed to each phage at three MOIs: 0.001, 1, and 10. Optical density (OD600) was collected in the Agilent LogPhase600 plate reader for 48 h at 37°C. Averages of the growth curve are graphed, with the gray area representing the standard deviation. (**D–F**) A one-way ANOVA was performed using the area under the curve (AUC), calculated with the Growthcurver package in R (59) after blank adjustment. For ANB28, the main effect of MOI is statistically significant and large (F [3, 32] = 58.90, *P* < 0.001; Eta2 = 0.85, 95% CI [0.76, 1.00]). Tukey’s HSD Test for multiple comparisons found that the bacterial control and an MOI 10 significantly differed from all other conditions. For SBP2Φ2, the main effect of MOI is statistically significant and large (F [3, 32] =172.45, *P* < 0.001; Eta2 = 0.94, 95% CI [0.91, 1.00]). For KB824, the main effect of MOI is statistically significant and large (F [3, 32] =51.31, *P* < 0.001; Eta2 = 0.83, 95% CI [0.73, 1.00]). For both KB824 and SBP2Φ2, Tukey’s HSD Test for multiple comparisons found that only the bacterial control significantly differed from all other conditions. Violin plots of AUC are shown with individual data points marked as dots. Data are represented by six growth curves: three biological replicates consisting of two technical replicates each, with bacterial controls assessed on each plate. Light purple represents a low MOI (0.001), purple represents a mid-MOI (1), dark purple represents a high MOI (10), and bacterial control is represented by black—significance level: *P* < 0.0001(****).

Growth curve analysis of each phage at MOIs of 0.001, 1, and 10 demonstrated that differences in the number of infecting virions for both KB824 and SBP2Φ2 did not significantly alter the dynamics of infecting host bacteria B28B, as measured in the area under the curve (AUC). KB824 delayed bacterial growth for 10 h in all MOI conditions (Fig. 5B and E). SBP2Φ2 suppressed bacterial growth for 18–20 h, with the two higher MOIs sharing similar growth patterns. The lower MOI condition moderately delayed bacterial growth, but no significant differences in the three MOI conditions were identified when AUC was evaluated (Fig. 5C and F). For ANB28, we observed that MOI 10 caused a significant reduction in overall bacterial growth as measured with the AUC. Surprisingly, for phage ANB28, MOI 0.001 trended longer in preventing resistant bacterial growth than MOI 1; however, there was no significant difference between the two MOIs as measured with AUC (Fig. 5A and D). These results indicate that under the tested conditions, the abundance of the three phages has little to no impact on phage predation against host bacteria B28B, as similar growth patterns emerge at the different MOI inputs.

### Establishment of a three-phage cocktail with distinct genomes and infection kinetics

**Fig 6:**
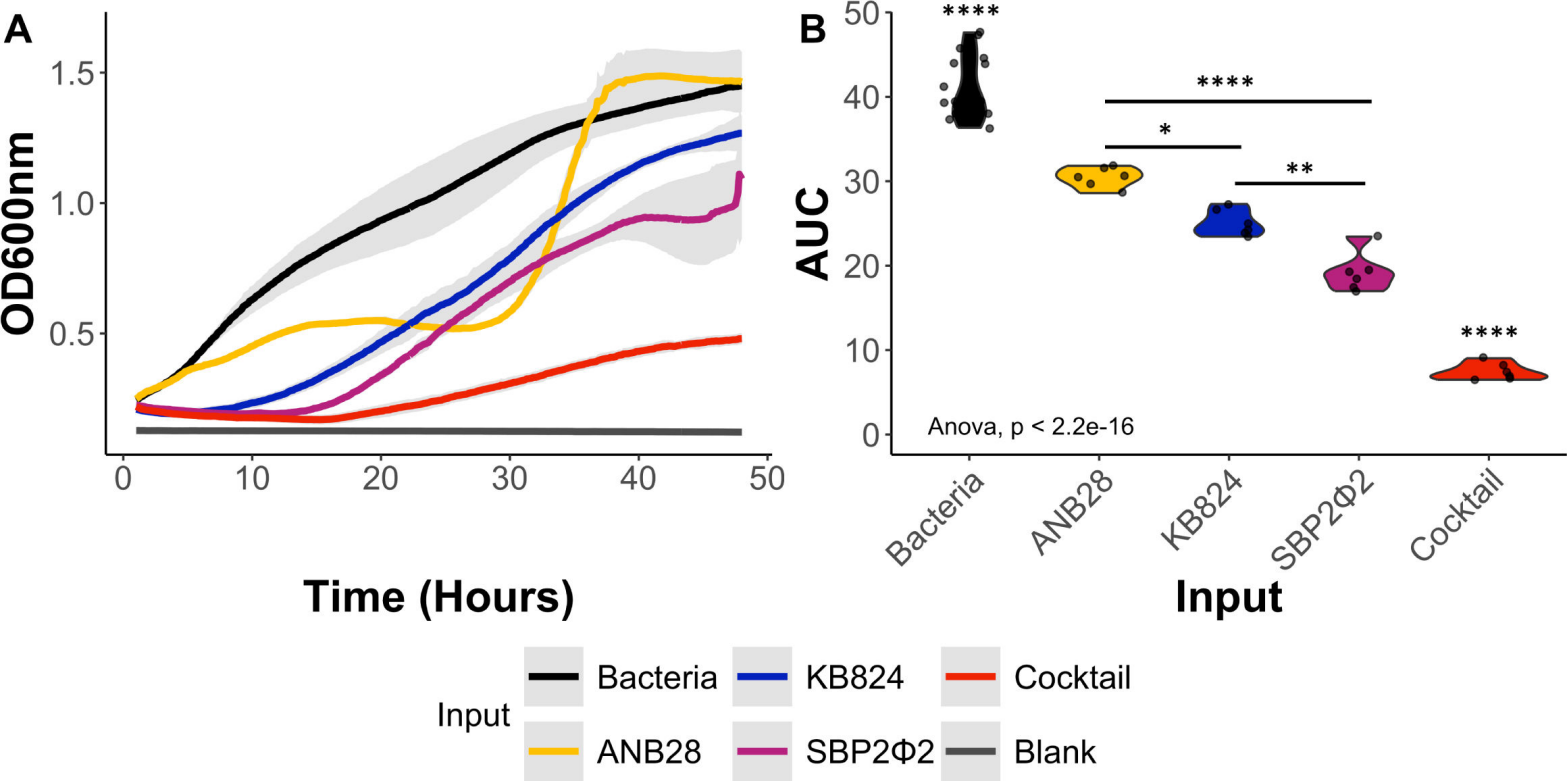
Impact of a three-phage cocktail against *S. maltophilia* host strain B28B. (**A**) Growth curve analysis of bacteria B28B against a three-phage cocktail consisting of phages ANB28, KB824, and SBP2Φ2. Log phase bacteria (OD600 0.1) were added to 96-well plates and exposed to individual phages and a three-phage cocktail at an MOI 1. Optical density (OD600) was collected with the Agilent LogPhase600 plate reader for 48 h at 37°C. Averages of the growth curves are graphed, with the gray area representing the standard deviation. (**B**) A one-way ANOVA was performed using the AUC after blank adjustment, calculated using the Growthcurver package in R (59). The main effect of input is statistically significant and large (F [4, 37] = 191.63, *P* < 0.001; Eta2 = 0.95, 95% CI [0.93, 1.00]). Tukey’s HSD Test for multiple comparisons found that all conditions were statistically different. Violin plots of AUC are shown with individual data points marked as dots. Data are an average of six growth curves comprising two technical replicates across three biological runs with bacterial controls assessed on each plate. Bacteria (black), ANB28 (yellow), KB824 (blue), SBP2Φ2 (magenta), and the three phage cocktail (red). Significance levels: *P* < 0.05 (*), *P* < 0.01 (**), and *P* < 0.0001(****).

We conducted an exploratory analysis of cocktail design using two different three-phage cocktails: one with phages sharing similar genomes (>97%) and the other with phages having distinct genomes (Fig. S5). The distinct three-phage cocktail was more effective at reducing bacterial growth in 11 *S*. *maltophilia* strains compared with a cocktail containing genetically similar phages. To further investigate, we conducted a growth curve analysis using host bacteria B28B with our three distinct phages in a cocktail and individual setting. The results indicated that the three-phage cocktail was optimal at suppressing host bacterial growth for an extended period (48 h) compared with individual phages (Fig. 6A). The AUC of the bacteria-only control was significantly elevated compared with all other conditions. At the same time, the AUC of the three-phage cocktail was significantly decreased compared with all other conditions. The AUC for individual phages varied in significance, with ANB28 and SBP2Φ2 showing the largest difference, followed by KB824 and SBP2Φ2, then ANB28 and KB824 (Fig. 6B). These results highlight that combining phages with distinct infection kinetics is an effective strategy for impacting the growth of multiple *S. maltophilia* strains and inhibiting bacterial growth in host bacteria B28B for an extended period.

**Fig 7:**
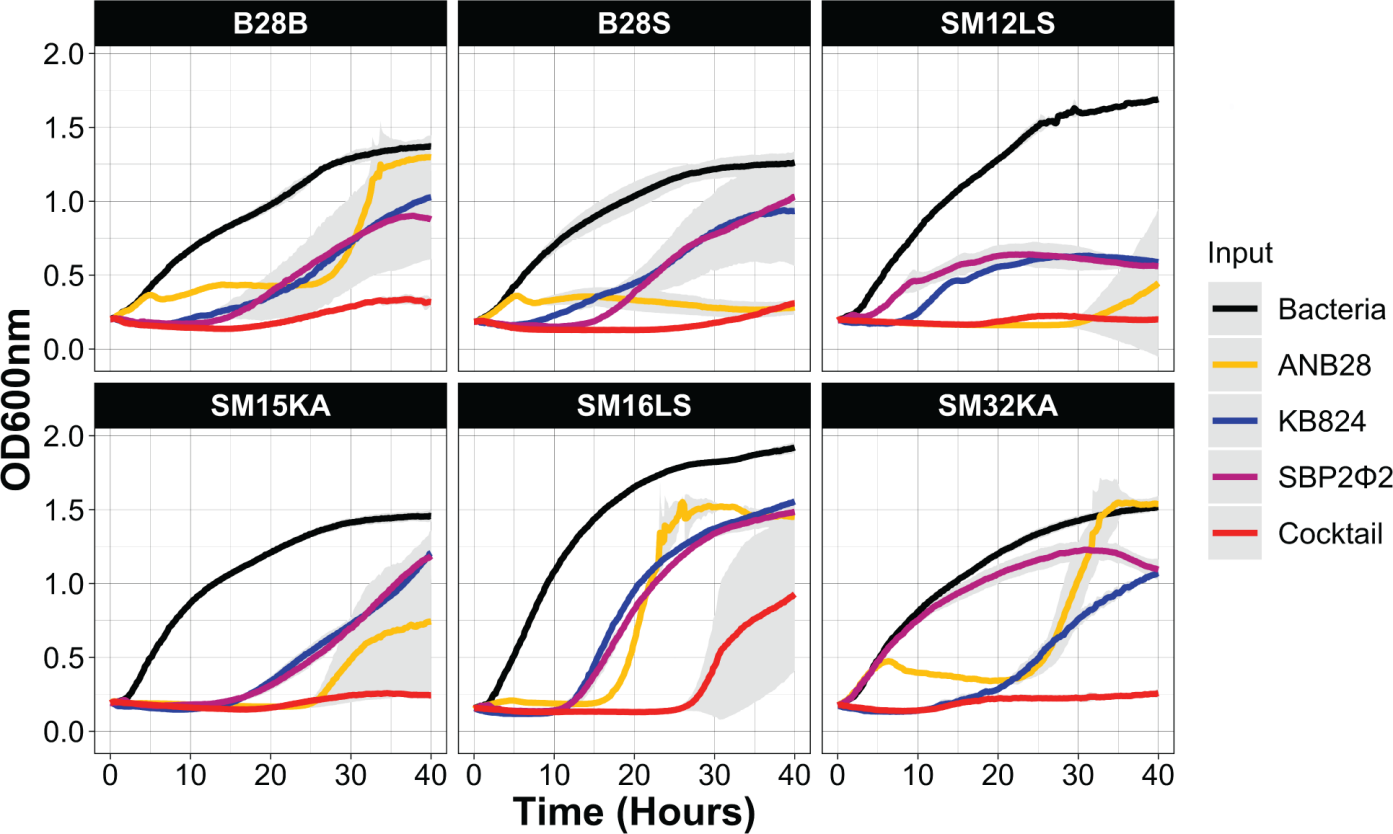
The impacts of a three-phage cocktail against six clinically relevant *S. maltophilia* strains. Strains were grown to a log phase (OD600 0.1) and exposed to phage ANB28, KB824, SBP2Φ2, and a three-phage cocktail comprising all three phages in a 96-well plate setup. Phages were exposed at MOI 1 based on the titer of host strain B28B. The results are presented as the average of three technical replicates, with the standard deviation denoted in gray. Bacteria (black), ANB28 (yellow), KB824 (blue), SBP2Φ2 (magenta), and the three phage cocktail (red).

Extensive host range analysis was performed with the three-phage cocktail and individual phages against 46 clinically relevant *S. maltophilia* strains at 37°C in BHI broth at an MOI 1 (based on host bacteria B28B) (Fig. S6). AUC was calculated for 12, 20, and 40 h after blank adjustment. The growth percentage was normalized to the bacteria-only condition to evaluate lytic activity in a strain-dependent manner using the following equation: [(1-(AUCcontrol - AUCphage)/AUCcontrol)*100]. The reduction in the red opacity indicates a reduction in bacterial growth; thus, lighter shades of red represent an increase in lytic phage activity. These results suggest that phage infectivity is highly selective. Additionally, we observed reduced bacterial growth when multiple phages can infect the same bacterial strain. Phage specificity is further supported by the interaction of phage KB824 against strains SM28LS and SM33KA, which demonstrated greater efficacy of the individual phage than the cocktail. This difference may be due to the number of KB824 phage particles, which is three times higher in the individual treatment than in the cocktail setting. Growth curves from six strains where the three-phage cocktail showed reduced bacterial growth at 40 h compared with individual phages were further analyzed (Fig. 7). The three-phage cocktail effectively suppressed bacterial growth of all six strains except for SM16LS, in which the cocktail caused a considerable delay. These results highlight the enhanced efficiency of a multi-phage cocktail, indicating a potential strategy for mitigating resistant bacterial growth.

### Investigating bacterial elements and lytic susceptibility in *S. maltophilia* strains

**Fig 8:**
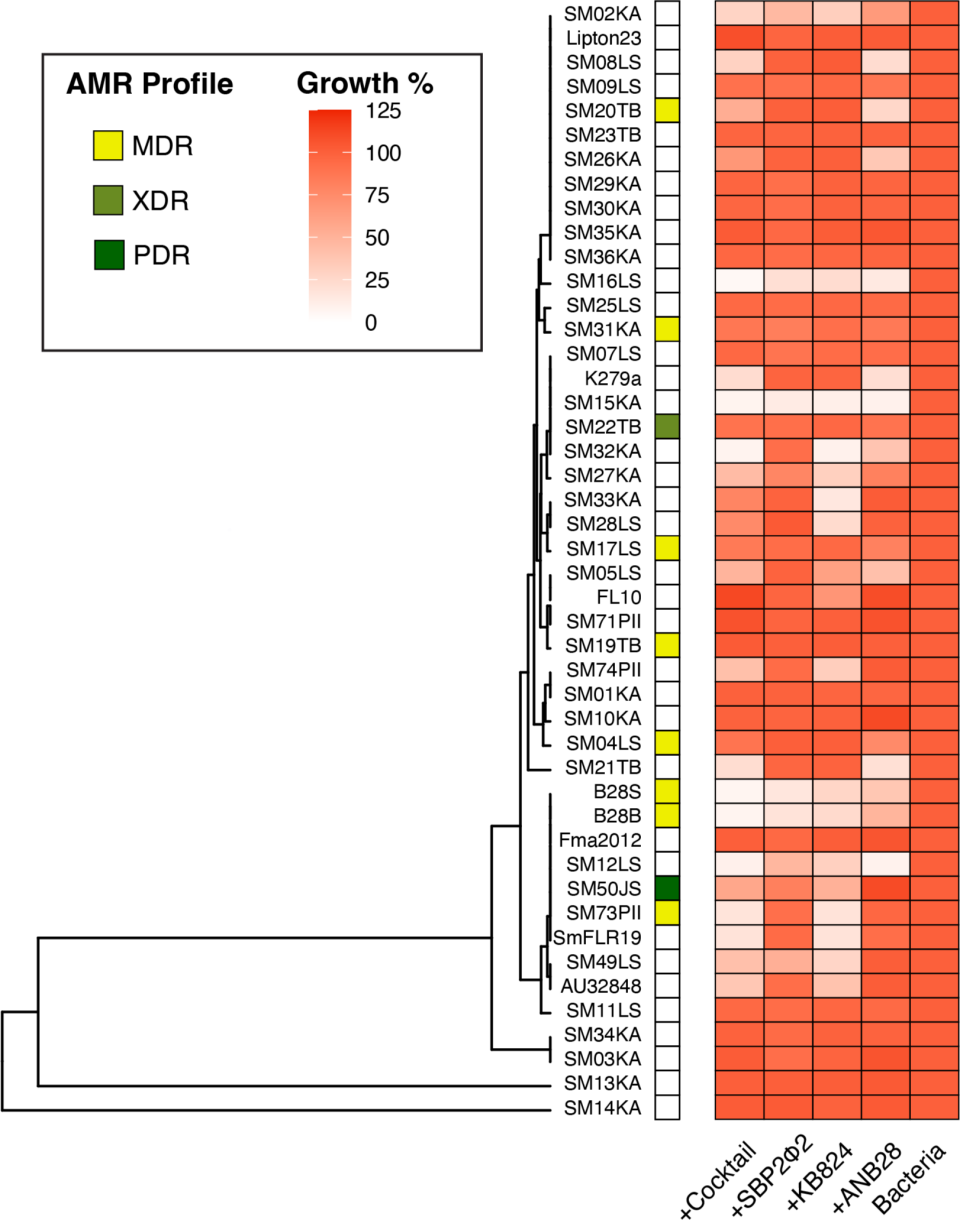
Phage host range analysis integrated with phylogenetic relationships and AMR profiles of tested *S. maltophilia* strains. Phylogenetic analysis was accomplished with Roary using an 80% percent identity threshold for BLASTp (60). The core alignment was processed with FastTree and visualized in R using hierarchical clustering with the “ape” and “ggtree” packages (61–63). Two strains were reclassified based on sequencing information and Kracken analysis: SM14KA as a *Serratia* spp. and SM13KA as *Achromobacter insolitus* (64). Strains names are listed on the left of the AMR profile with designation for MDR in light green, XDR in green, and PDR in forest green (based on sensitivity to ceftazidime, minocycline, trimethoprim-sulfamethoxazole, and levofloxacin). The heatmap on the right shows the lytic profiles of phage ANB28, KB824, SBP2Φ2, and a three-phage cocktail (MOI 1, based on host strain B28B). The AUC was calculated at 20 h and normalized to bacterial growth per strain. The color gradient indicates growth relative to control (darker red indicates growth, and lighter shades indicate phage lysis).

The AUC for 20 h, which was used to infer the host ranges for phages ANB28, KB824, SBP2Φ2, and the three phage cocktail, was integrated with the AMR profile and organized by phylogenetic clades to evaluate the potential associations between phage susceptibility and bacteria phylogeny (Fig. 8). Our *S. maltophilia* bacteria collection contained eight MDR strains (resistant to at least one antibiotic in three or more categories), including the host bacteria B28B investigated in this study, one XDR strain (resistant to all but one or two antimicrobial categories), and one PDR strain (resistant to all antibiotic agents). Additionally, SM13KA and SM14KA were re-identified based on sequencing information as *Achromobacter insolitus* and *Serratia spp*., respectively. Interestingly, in strains within the clade containing host bacteria B28B (from strain B28S to SM11LS), we see increased sensitivity to phage KB824, with eight of 10 strains showing susceptibility. However, the top clade (from strain SM02KA to SM36KA) revealed that only four of 11 strains were susceptible to phage ANB28. These results highlight the complexity of bacteria-phage interactions; the data may suggest potential relationships between phage susceptibility and bacterial phylogeny. However, this relationship was not universally observed and must be further investigated.

## DISCUSSION

Phage therapy is becoming increasingly important as MDR bacterial infections continue to rise. The recalcitrant opportunistic pathogen *S. maltophilia*, which possesses intrinsic AMR mechanisms that compromise current treatment options, is an attractive target for phage therapy. Despite its promise, the development of phage resistance and the complex network of phage-bacteria interactions present a hurdle that phage therapy must overcome to become widely implemented. To address some of these challenges and build upon our understanding of phage-host dynamics, we investigated the genetics, host range, and infection kinetics of three distinct phages and examined their capacity to reduce bacterial growth when combined into a cocktail.

We isolated and analyzed 18 *S*. *maltophilia* phages and identified three genetically distinct clusters. Based on this genetic analysis, we selected three representative phages—ANB28, KB824, and SBP2Φ2—for further evaluation. ANB28 stood alone as the only phage within its cluster, and BLASTn analysis revealed low similarities between ANB28 and any known phage. We conducted phylogenetic analysis with 27 previously identified *S. maltophilia* phages, and ANB28 and SBP2Φ2 showed significant divergence. However, this analysis contains only a snapshot of phage diversity due to cultivation bias (our phage hunting methods and choice of host strains likely selected for a small subset of phages); further development of our phage library is critical for a thorough understanding of *S. maltophilia* phage diversity.

Impacts of phage predation against host bacteria B28B were not significantly altered by changes in phage abundance, as noted in our MOI growth curve, with the exception of the highest MOI condition for phage ANB28. High MOIs have been associated with lysis from without, whereas low MOIs could impact phage penetration to host bacteria, demonstrating the complexity of phage abundance (65). A previous study has shown that MOI does not significantly impact the growth dynamics between the phage and its host bacteria when evaluated against other factors, such as phage species (66). Additionally, in one study, MOI 10 and 100 were able to eliminate septic infections in mice with a 100% survival rate; however, the lower MOI (MOI 1) only achieved 87.5%, highlighting the importance of phage dosing (67).

Our three phages are distinguishable through their plaque morphology, host range, and genetic makeup. Differences in adsorption rate, latent period, and burst size when infecting bacterial host B28B help us to understand phage-bacteria interactions, underlining that each phage carries out a unique replication program with distinct kinetics. Our isolated siphoviruses, ANB28 and SBP2Φ2, had latent phases of 90 and 80 min, respectively, consistent with other previously isolated *S. maltophilia* siphoviruses, AXL1 (90 min) and StM171 (130 min) (42, 45). KB824, our podovirus, exhibited a short latent phase of 30 min, aligning well with latent phases of previously characterized *S. maltophilia* podoviruses A1432, BUCT548, and BUCT598 (41, 68, 69). Thus, replication strategies may correlate to phage morphology even with differences in experimental conditions such as starting MOI, media conditions, and researchers. Knowing critical checkpoints in phage predation offers opportunities to investigate the molecular processes in phage-infected bacteria. For example, previous research in *Pseudomonas aeruginosa* showed that phage phiKZ and YuA mediate time-dependent alteration in bacterial metabolism, highlighting the interplay between phage auxiliary metabolic genes and bacteria physiology (70). Such deep insights can help identify potential vulnerabilities of the host bacteria, which subsequently can be leveraged to design more informed and effective phage therapy strategies.

Additionally, we showed the impacts of a multi-phage cocktail on a susceptible host bacteria B28B. We provided supporting evidence that genetically distinct phages with unique infection kinetics enhance suppression of bacterial growth and may give the phages an upper hand in the evolutionary arms race between the phage and its bacterial host. Our preliminary experiments on phage cocktail design suggest that a genetically diverse cocktail was more effective at reducing bacterial growth than a cocktail comprising genetically similar phages with 11 *S*. *maltophilia* strains (Fig. S5). One caveat regarding phage cocktails is their potential for enhanced immunogenic responses, such as the production of neutralizing antibodies that could render phage treatment ineffective (71). Single-phage therapeutics have proven successful, especially when combined with antibiotics and used in conjunction with treatment approaches that prevent or overcome resistance, such as cycling (72). Although this approach can potentially increase bacterial killing, the complex strategy requires real-time data analysis for isolated bacterial cultures, which could delay treatment. With frequent therapeutic failures in the treatment of recalcitrant *S. maltophilia* infections with antibiotics, phages and phage cocktails could become a critical, life-saving strategy. Our research demonstrates the potential benefits of considering genomic diversity when tailoring phage cocktails to specific bacterial strains, enhancing the eradication of the targeted bacteria.

Phage susceptibility and bacterial phylogeny were also investigated, and although trends were noted, the association was not universal, highlighting the complexity of phage-bacteria interactions. At 20 h, KB824 (podovirus) infected 18 of 46 strains, whereas SBP2Φ2 and ANB28 had narrower host ranges, infecting seven and 15 strains, respectively. These data are consistent with those of previous research showing that *S. maltophilia* siphoviridae have a narrow host range (35, 43). In our study, six of 46 strains demonstrated prolonged suppression of bacterial growth with our phage cocktail at an MOI of 1. The details of the conditions have a big impact, as several recent studies have shown; for example, a phage cocktail against a diverse cohort of clinical *P. aeruginosa* isolates was more broadly effective, even in biofilm populations, but at much higher MOIs (73). Finally, although our study shows the efficacy of a three-phage cocktail against rapidly growing bacteria under laboratory conditions, further research should examine the phage infection dynamics across bacterial growth stages, including *in vivo* infections that harbor bacteria in multiple phases of growth, including biofilms and polymicrobial communities (30, 74).

In summary, our results highlight the distinct infection kinetics of each phage in our cocktail and suggest a cooperative kinetic interaction that helps control bacterial growth. The focus on kinetics provides a new perspective on previous data showing that cocktails comprised of genetically distinct phages effectively eradicate MDR bacteria (24, 27). Further studies relating to phage kinetics and genetics are vital and may play an important role in future cocktail design. Thus, validating these results could accelerate the development and optimization of phage cocktails. Establishing routine phage therapy would help mitigate MDR-associated mortality rates and reduce human suffering.

## MATERIALS AND METHODS

### Bacterial cultures

The bacterial strains used in this study are listed in Table S1. *S. maltophilia* strain B28B was isolated from an oncology patient at University of California, San Diego (UCSD) in July 2020. B28B was grown in brain heart infusion broth (BHI; Research Products International) at 37°C on a 200 rpm shaker. Glycerol stocks were made at a final glycerol concentration of 25%. Bacteria were grown by streaking from glycerol stocks onto BHI plates and incubated at 37°C for 18–20 h. For experiments and assays, isolated colonies were grown in overnight broth culture; the next day, a 1:10 or 1:20 dilution into BHI was placed at 37°C on a 200 rpm shaker to achieve a log phase at OD600 of 0.3 or 0.1, respectively. Antimicrobial sensitivity testing (AST) for *S. maltophilia* strains was performed at UCSD in the clinical microbiology department using micro broth dilution on the BD Phoenix. Standard Kirby Bauer Tests were performed for all others, and the zone of inhibition was measured using ImageJ after calibrating with a known distance (75). Antibiotics that were used to determine AMR profile include ceftadimize (CAZ; Oxoid Cat. CT0412B), levofloxacin (LEV; Oxoid Cat. CT1587B), minocycline (MH; Oxoid Cat. CT0030B), and trimethoprim-sulfamethoxazole (SXT; Cat. CT0052B).

### Phage lysate, titering, and plaque morphology

The phage isolates used in this study are listed in Table S2. Phage propagation was based on Bonilla et al. (76). Phage lysates were stored with a final concentration of 10% glycerol at −80°C. Phage titering was done every 2 weeks and recorded over time based on harvest date, correlating to a specific lot number. For plaque morphology and phage titering, serial dilutions of phage lysates were used to achieve a countable plaque number. Plating consisted of 10 μL of the diluted lysate against 100 μL of log phase host bacteria B28B using a BHI soft agar overlay incubated at 37°C for 18–20 h. Three technical replicates were averaged to calculate the PFU/mL of the stock concentration of a lysate or digitally imaged for plaque morphology. The phage titer of KB824 for temperature assessment was conducted similarly. Duplicate plates were made; one set was incubated at 37°C, whereas the other set was incubated at room temperature for 18–20 h and then scanned using the EPSON Perfection V600 Photo Scanner.

Phage DNA was extracted from high-titer stocks using a QIAamp UltraSens Virus kit (Qiagen, Cat. 53706) per the manufacturer’s instructions. Before performing the DNA extraction, all phages were treated with 2 μL of RNAase A (50,000 U/mL, New England BioLabs, Cat. M02403S) and 50 μL of NEB buffer, followed by 5 μL of DNAase I (2,000 U/mL, New England BioLabs, Cat. M0303S). The samples were then treated with 50 μL of NEB buffer for a 30 min incubation at 37°C, followed by a 10 min incubation at 74°C to inactive the enzymes. The extracted DNA was quantified using a Qubit dsDNA High Sensitivity Assay Kit (Invitrogen, Cat. Q32851), and library preparation was done using the Nextera XT DNA LP kit (Illumina). Sequencing was performed on Illumina’s iSeq100 using a paired-end approach (2*150  bp). Raw Illumina reads were uploaded to the University of California, Irvine’s High-Performance Community Computing Cluster (HPC3) and cleaned with “bbduck,” and duplicates were removed with “dedup,” both from bbtool (50). Human contamination was removed with Bowtie2 v2.4.1 (77). Reads were assembled with unicycler (51), checked for quality with QUAST, (78) and annotated with RASTtk (52). Coverage analysis was used to identify the contig of interest if sequencing resulted in multiple contigs. FASTA files were concatenated and uploaded to the VIRIDIC server for the VIRIDIC analysis (79), whereas CheckV analysis was run in the command line (80). VIRIDIC analysis was also conducted for our distinct and similar three-phage cocktails. Phage therapy candidacy screening of FASTA file for AMR genes and toxin-encoding genes was accomplished using the CARD database (55) and TAfinder (56), respectively. Genome maps were visualized in Geneious Prime with GenBank files, and manual checks were performed for integrase genes, hypothetical proteins (HP), transfer RNAs (tRNAs), open reading frames (ORFs), GC%, and genome length (57). Coverage plots were performed by mapping clean reads to a Bowtie2 database for each phage FASTA file (77). Samtools was then used for read counts (81), whereas data visualization was done in R (82). BLASTn analysis was conducted on each phage, and the top four hits were identified, compiled, and sorted to determine the comparative genomics of each phage cluster using R (82). These top BLASTn hits for each phage cluster, along with all of our *S. maltophilia* phages, were then used for comparative genomics in three different groups: Cluster 1, Cluster 2, and Cluster 3. GenBank files were analyzed in Anvi’o using the ANI% option (“anvi-compute-genome-similarity”) and visualized with their established interface (53). The output of ANI% for all our phages was visualized in R (82). Phylogenetic analysis was performed in VIPtree (54) using FASTA files from a compiled list of *S. maltophilia* phages collected from the literature source (35).

### Bacterial sequencing and bioinformatics

For bacterial DNA extractions, overnight bacterial growth was extracted using either the Invitrogen DNA Mini Kit (Invitrogen, Cat. K182002) or DNeasy Blood and Tissue Kit (Qiagen, Cat. 69506) per the manufacturer’s instructions. Library preparation and sequencing were performed at the Microbial Genome Sequencing Center (MIGs) or the UCI Genomics Research and Technology Hub (GRT-Hub) (Table S1). In-house library preparation was done using the Nextera DNA Flex Library Prep Kit (Cat. 20018705) following a low-volume protocol (83). The only modification was using Phusion DNA Polymerase (ThermoScientific Cat. F-549L) for Ready Mix HiFi Kappa (Roche Life Science Cat. KK2602). Sequencing was performed on Illumina’s iSeq100 using a paired-end approach (2*150  bp). For MIGs, libraries were prepared using the Illumina DNA Prep kit and IDT 10 bp UDI indices and sequenced on an Illumina NextSeq 2000, producing 2 × 151 bp reads. Additionally, FASTQ files for four strains were pulled from either NCBI or Bacterial and Viral Bioinformatics Resource Center (BV-BRC) using the accession number noted (Table S1). Raw reads were uploaded to HPC3, trimmed, and cleaned with “bbduck,” and duplicates were removed with “dedup,” both from bbtool (50). Reads were assembled with unicycler (51) and checked for quality with QUAST (78) and CheckM (84). Two strains were reclassified based on sequencing information and Kracken analysis (64). Phylogenetic analysis was accomplished with Roary set to 80% for the minimum percentage identity for BLASTp (60). FastTree was then used for core genome alignment, and data visualization was accomplished with R using both “ape” and “ggtree” (61–63).

### Efficiency of plating

B28B was grown to log phase (OD600 0.3) in BHI broth. Molten agar overlays of 4.9 mL were performed on square Petri plates (VWR Cat. 60872–310) using 140 μL of bacteria culture and allowed to solidify at room temperature for 40 min. Phage stocks were processed to a 10^7^ PFU/mL titer, and serial dilutions were made to achieve 1 × 10^6^, 1 × 10^5^, 1 × 10^4^, and 1 × 10^3^ PFU/mL titer. Aliquots of the phage dilution were added to the bacterial lawn in a 3 µL volume and allowed to dry. Plates were incubated at 37°C for 18–20 h before being scored for lysis based on a published protocol (85). Each phage dilution was run in technical duplicate against 13 clinically relevant *S. maltophilia* strains.

### Phage morphology by electron microscopy

After phage propagation, to observe virion morphology, the samples were negatively stained using established procedures (58) (briefly summarized here) and imaged by electron microscopy. 200-mesh Gilder copper grids (Ted Pella) were carbon-coated in-house, and 0.75% Uranyl Formate stain was freshly prepared. Grids were negatively glow discharged using a PELCO easiGlow (Ted Pella) prior to staining. Samples were stained as-is, and a dilution series was used to avoid potential overpacking. Three microliters of each sample were applied to a grid and allowed to adsorb for 10 s before the excess liquid was removed using filter paper, washed twice with Milli-Q water, stained using 0.75% Uranyl Formate, and allowed to air dry. All grids were imaged, and data were collected using a JEOL JEM-2100F transmission electron microscope equipped with a Gatan OneView 4k × 4k camera. Scale bars for the representative images that appear in Fig. 3A through C were added and contrast was enhanced using ImageJ (75).

### Rate of attachment

The rate of attachment was determined based on the method described in Kropinski et al. (17), with minor modifications. B28B was grown to a log phase (OD600 0.3) in BHI broth. The adsorbance flask (9 mL of bacteria) and media flask (9 mL of BHI) were equilibrated for 5 min at 37°C and shaken at 200 rpm (Entech Instruments 5600 SPEU) before 1 × 10^5^ PFU were added to both flasks (t = 0). Vials of 50 µL of CHCl_3_ and 950 µL of BHI were chilled for 10 min before adding 50 µL of bacteria-phage mixture. Sampling was performed every minute, vortexed, and placed on ice. Controls were sampled and processed after the 10 min experimental samples were obtained, as previously described for the experimental conditions. The molten overlay was performed chronologically for each time point and the two controls. Petri plates solidified at room temperature (RT) for 40 min and then incubated at 37°C for 18–20 h. Data of absolute PFUs were recorded and converted into percentages of free phage by dividing the average control value. Each phage isolate was performed against three biological replicates of host bacteria, B28B.

### One-step growth curve

This protocol was performed with minor adjustments as described previously (18). B28B was grown to log phase (OD600 0.3) in BHI broth. An adsorption flask was prepared with 900 µL of bacteria, whereas the dilution flasks (10^−2^ flask and 10^−4^ flask) were prepared with 9.9 mL of fresh BHI. All flasks were placed on a shaker (~200 rpm) to equilibrate to 37°C (Entech Instruments 5600 SPEU). Phage was added to the adsorption flask at an MOI of 0.001 in a 100 µL volume and mixed well. Immediately afterward, 100 µL was taken from the adsorption flask, added to the 10^−2^ flask, and mixed well; this process was repeated from the 10^−2^ flask to the 10^−4^ flask. For phage ANB28, a 10^−3^ flask was prepared. Directly following, 2 mL of the 10^−4^ flask (for phage ANB28, 10^−3^ flask) was removed and added to a microcentrifuge tube containing 50 μL chilled CHCl_3_. At specific time points, aliquots of either 500 µL, 250 µL, 100 µL, or 50 µL were taken from the diluted flask, which was then used in the molten agar overlay with host bacteria to achieve countable plaques. Upon completion of the phage-bacteria sampling, either 500 µL, 250 µL, 100 µL, or a combination of the two were taken from the CHCl_3_-treated control and processed, as previously stated. Petri plates were allowed to solidify at RT and then incubated at 37°C for 18–20 h. Absolute PFUs were counted and calculated into PFU/mL with averaged control values of two duplicates subtracted from each data point and then graphed.

### Three-phage cocktail design - similar versus distinct phages

Eleven *S. maltophilia* strains were grown to log-phase (OD600 0.3) in BHI broth and plated in designated wells on a 96-well plate. The strain list included: B28B, B28S, K279a, SM12LS, SM15KA, SM17LS, SM26KA, SM27KA, SM49LS, SM71PII, and FL10. Media and bacteria aliquots were added at 180 μL, whereas phage lysate or SM buffer was added at 20 μL aliquots. Phage cocktails were added at an MOI of 1 based on host bacteria, B28B, and each cocktail incorporated a third of each phage. The distinct phage cocktail was composed of ANB28, KB824, and SBP2Φ2. A similar phage cocktail was composed of KB824, SBP4Φ1, and SBP5Φ1. Plates were run on a Molecular Device Plate Reader for 48 h at 37°C. Data were analyzed in R using “dplyr” for data wrangling, “gcplyr” for AUC calculation, and “ggplot2” for plot visualization (82, 86–88). A one-way ANOVA was used to determine if the AUC for each phage cocktail differed, with a follow-up post hoc test performed to confirm which conditions were statistically different from each other.

### MOI and cocktail growth curves

B28B was grown to log phase (OD600 0.1) in BHI broth. A 96-well plate with a water perimeter (~200 µL/well) to reduce experiment evaporation was used. Media controls and bacterial aliquots of 180 µL were placed into designated wells. Phage lysates were diluted in SM buffer to achieve an MOI of 0.001, 1, and 10 in a 20 µL aliquot. The MOIs were held constant for the three-phage cocktail, incorporating a third of each phage. Either phage dilutions or SM buffer were placed in the designated wells. Plates were run on the Agilent LogPhase600 for 48 h at 37°C. Data were graphed in R using “dplyr” and “ggplot2” to assess for bacterial contamination in a 96-well plate layout (86, 87). AUC was determined with the Growthcurver package (59). Statistics were conducted in R (82) using a one-way ANOVA to determine if the AUC for each phage input differed. A post hoc test was performed to identify which conditions and phages were statistically different.

### Host range growth curves

All *S. maltophilia* strains were grown to a log phase (OD600 0.1) in BHI broth. Each *S. maltophilia* strain was exposed to ANB28, KB824, SBP2Φ2, and a combination of the three phages at an MOI of 1 based on the host strain B28B in technical triplicates. A 96-well plate with water (~200 µL/well) in the top and bottom rows was used to reduce evaporation. Media controls and bacterial aliquots of 180 µL were placed into designated wells. Phage lysates were diluted in SM buffer to achieve a 20 µL aliquot, and either phage dilutions or SM buffer was placed into the designated wells. An MOI of 1 was held constant for the three-phage cocktail, incorporating a third of each phage. Plates were run on the Agilent LogPhase600 for 48 h at 37°C. Data were graphed in R using “dplyr” and “ggplot2” to assess for bacterial contamination in a 96-well plate layout (86, 87). AUC was calculated using “gcplyr,” and technical replicates were averaged after removing the blank (88). Growth percentage was calculated using the following equation: Growth% = [1−(Average Bacteria only AUC − Average Phage AUC)/Average Bacteria only AUC]*100, and data were visualized with heatmaps using “pheatmap” (89).

## Data Availability

The code for analyzing and making figures is available at https://github.com/Amonsiba/STM_phage_cocktail. Raw phage sequencing data have been uploaded to the SRA under BioProject PRJNA1121625, and raw bacterial sequencing data are under BioProject PRJNA1182746. NCBI accession numbers for phages are located in Table S2.

## SUPPLEMENTAL MATERIAL

Supplemental figures aac.01162-24-s0001.docx Figures S1 to S6.

Table S1 aac.01162-24-s0002.xlsx Bacterial isolate names, accession numbers, or sequencing center location.

Table S2 aac.01162-24-s0003.xlsx Phage details, including isolation source, host strain, and GenBank accession ID.

Table S3 aac.01162-24-s0004.xlsx CheckV analysis to assess quality control of phage genomes isolated in this study.

Table S4 aac.01162-24-s0005.xls BLASTn analysis of each phage isolated in this study.

Table S5 aac.01162-24-s0006.xlsx CheckM analysis to assess quality control of bacterial genomes used in this study.

ASM does not own the copyrights to Supplemental Material that may be linked to, or accessed through, an article. The authors have granted ASM a non-exclusive, world-wide license to publish the Supplemental Material files. Please contact the corresponding author directly for reuse.
