# Supplementary material for: Enhanced suppression of Stenotrophomonas maltophilia by a three-phage cocktail: genomic insights and kinetic profiling
Source: Antimicrob Agents Chemother. 2025 Jan 22;69(3):e01162-24. doi: 10.1128/aac.01162-24 (PMC11881566; doi:10.1128/aac.01162-24)
Supplement: Supplemental figures — Figures S1 to S6. [file aac.01162-24-s0001.docx]

**SUPPLEMENTAL FIGURE**


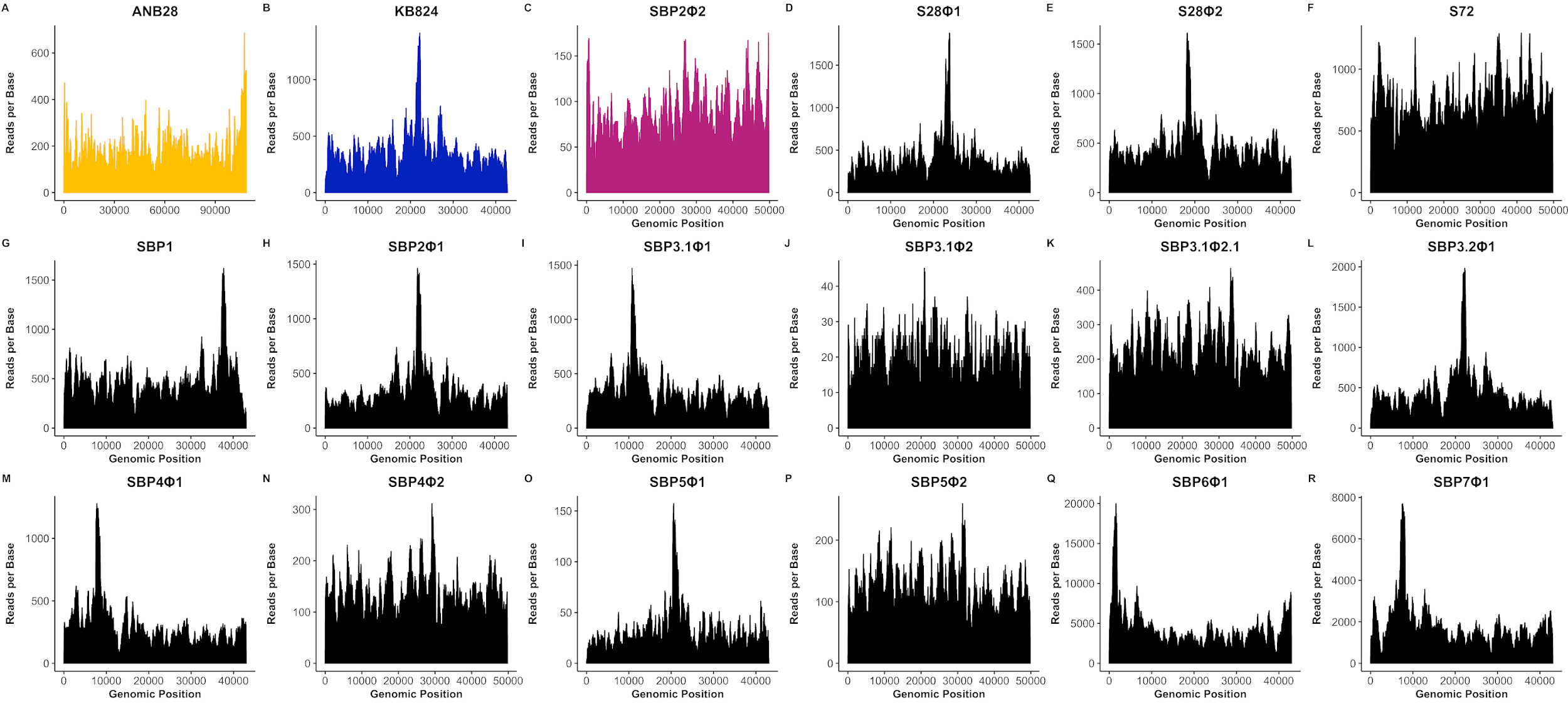


**Supplemental Figure S1. Read Coverage of Phage Contigs.** (A-R) Raw reads were cleaned, trimmed, and deduplicated with bbtools (63), while human contamination was removed with bowtie2 (64). Contig databases and read mapping were accomplished with bowtie2. Read counts were performed with samtools (73), and data visualization was accomplished in R (74). Selected phages are highlighted in color: ANB28 (yellow), KB824 (blue), and SBP2Φ2 (magenta).


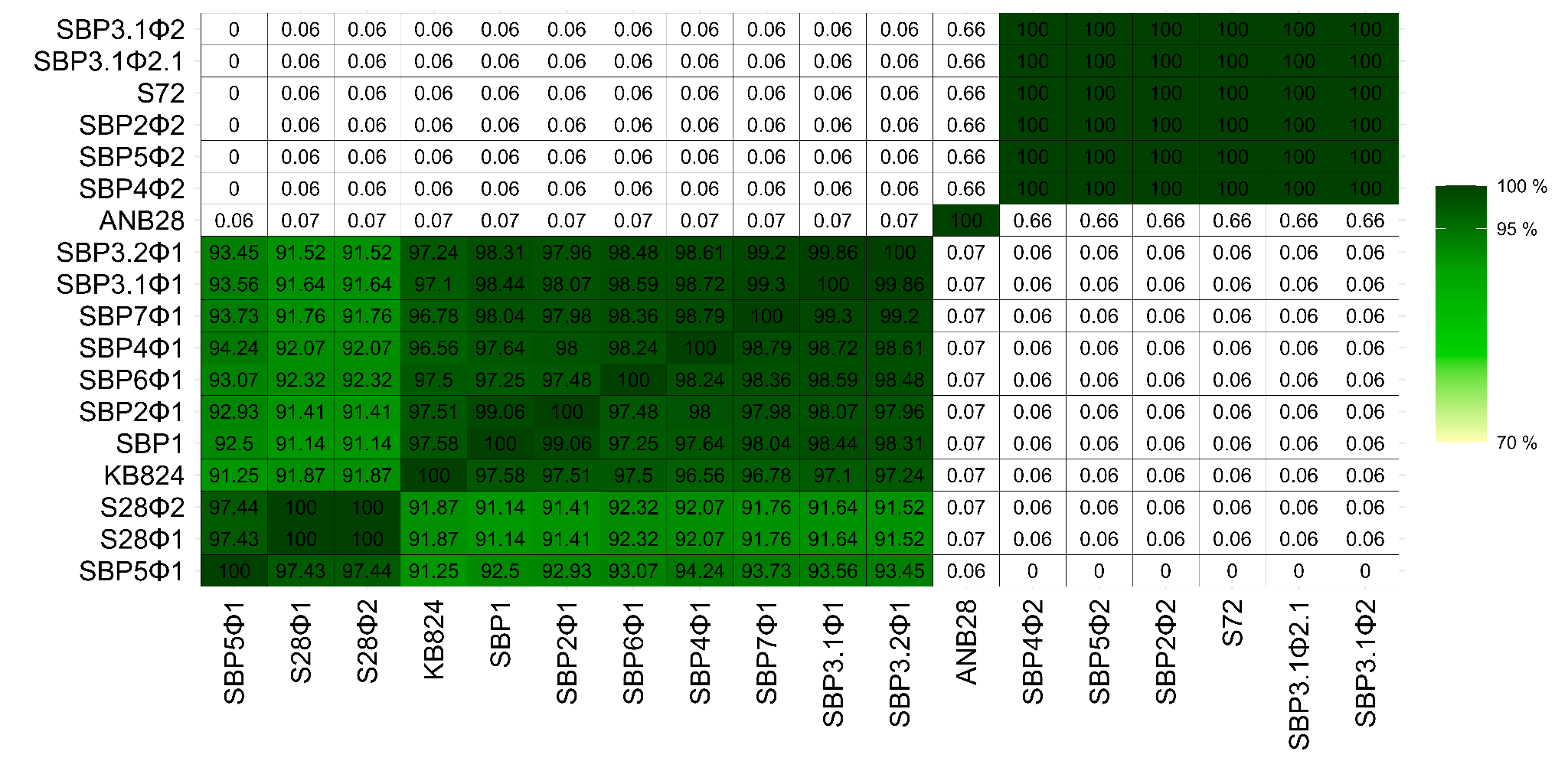


**Supplemental Figure S2. VIRIDIC Analysis of fasta files for *S. maltophilia* phages.** All fasta files were renamed to human readable sample identification, concatenated into one file, and uploaded to the VIRIDIC server (68). Data visualization was performed in R (74).


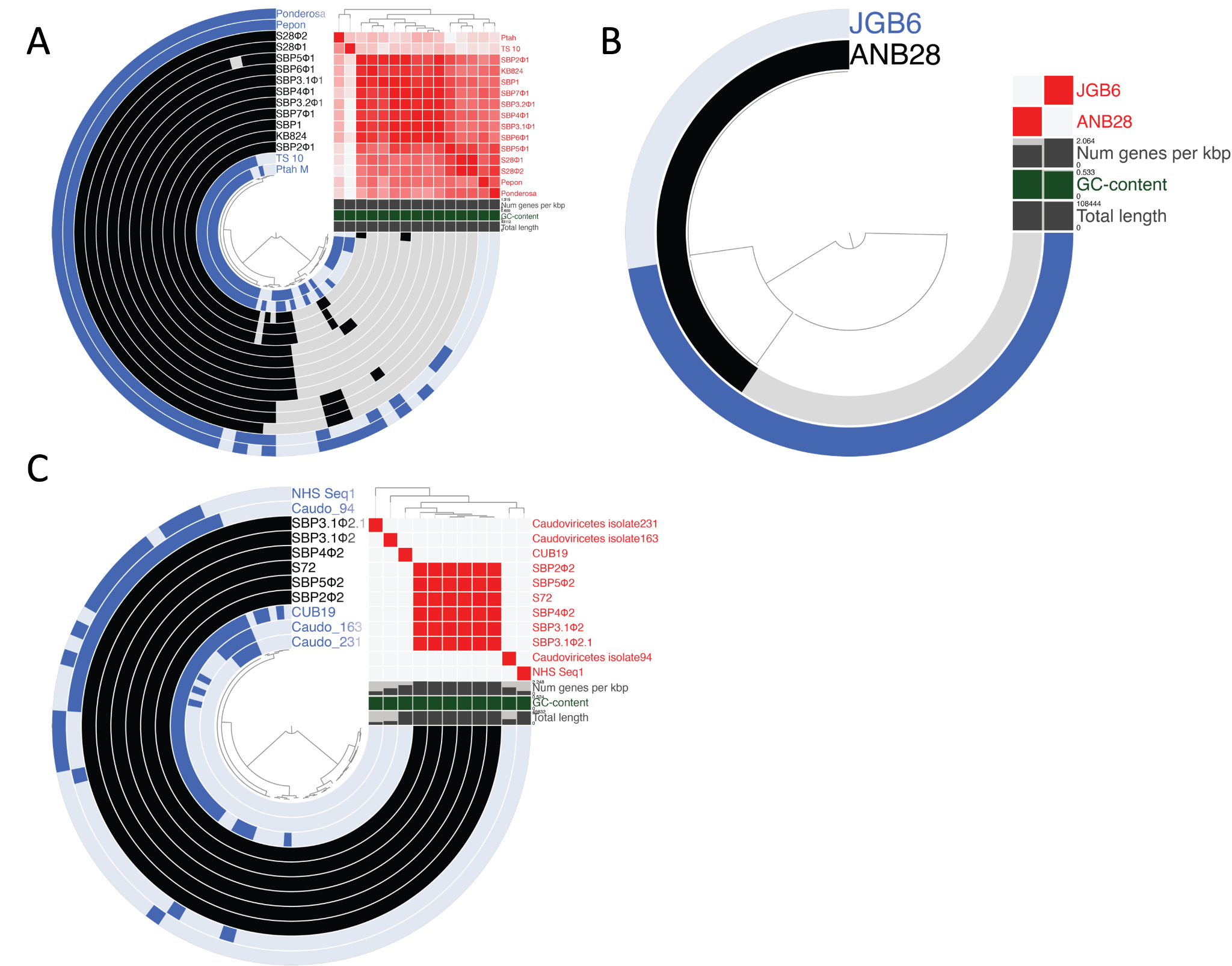


**Supplemental Figure S3.** **Comparative Genomics of *S. maltophilia* phages using ANVI’O**. GenBank files were processed into contig databases with external gene calls and functional annotations. Pangenomic analysis was performed first by establishing a genome storage and processing the data with the “anvi-compute-genome-similarity” option to calculate the Average Nucleotide Identity Percentage (ANI%) (75). Characterization of the genomes includes the number of genes per kbp (gray), GC content (green), and total length (gray). Gene clusters are organized by presence and absence and represented by black or darker blue sections. (**A**) Cluster 1 (based on Figure 1) represents proviruses and the top 4 phage genomes identified in the BLASTn analysis. (**B**) ANB28 and its closest phage genome from BLASTn analysis. (**C**) Cluster 3 (based on Figure 1) represents siphoviriuses and the top 5 BLASTn phage genomes identified in BLASTn analysis. Phages identified in the BLASTn analysis are highlighted in blue, while *S. maltophilia* phages isolated in this study are black/gray.

**
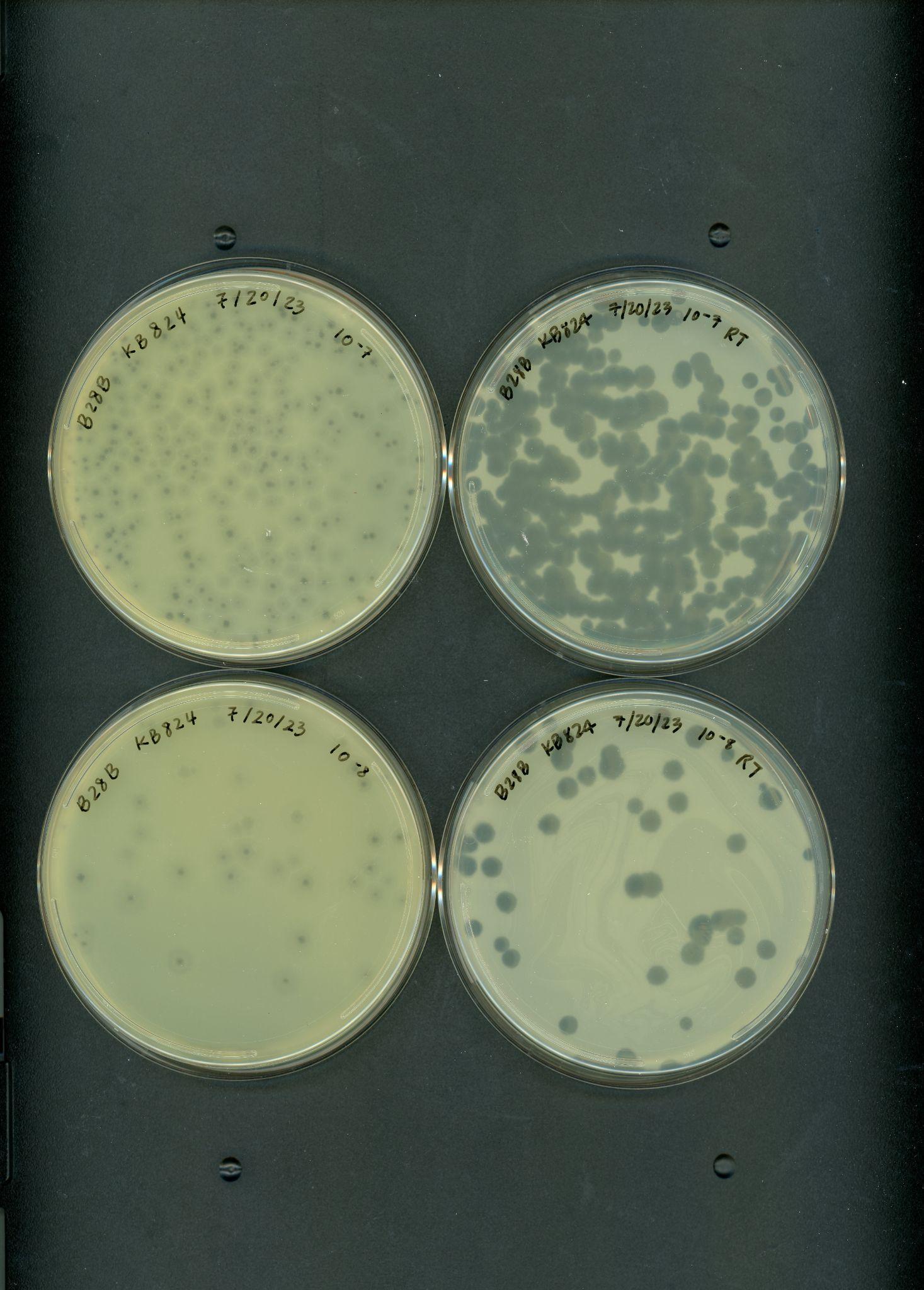
**

**Supplemental Figure S4.** **KB824 Titer at Room Temperature vs 37°C**. Log phase host bacteria B28B was exposed to KB824 stock dilutions of 10^-7^ and 10^-8^ in aliquots of 10 uLs. Phage-Bacteria mixtures were processed with a soft agar overlay, solidified for 40 minutes, and then incubated for 18-20 hours at either room temperature (~20°C) or 37°C. Plates were then scanned using EPSON Perfection V600 Photo to illustrate the difference in lytic activity at the two temperatures.

**
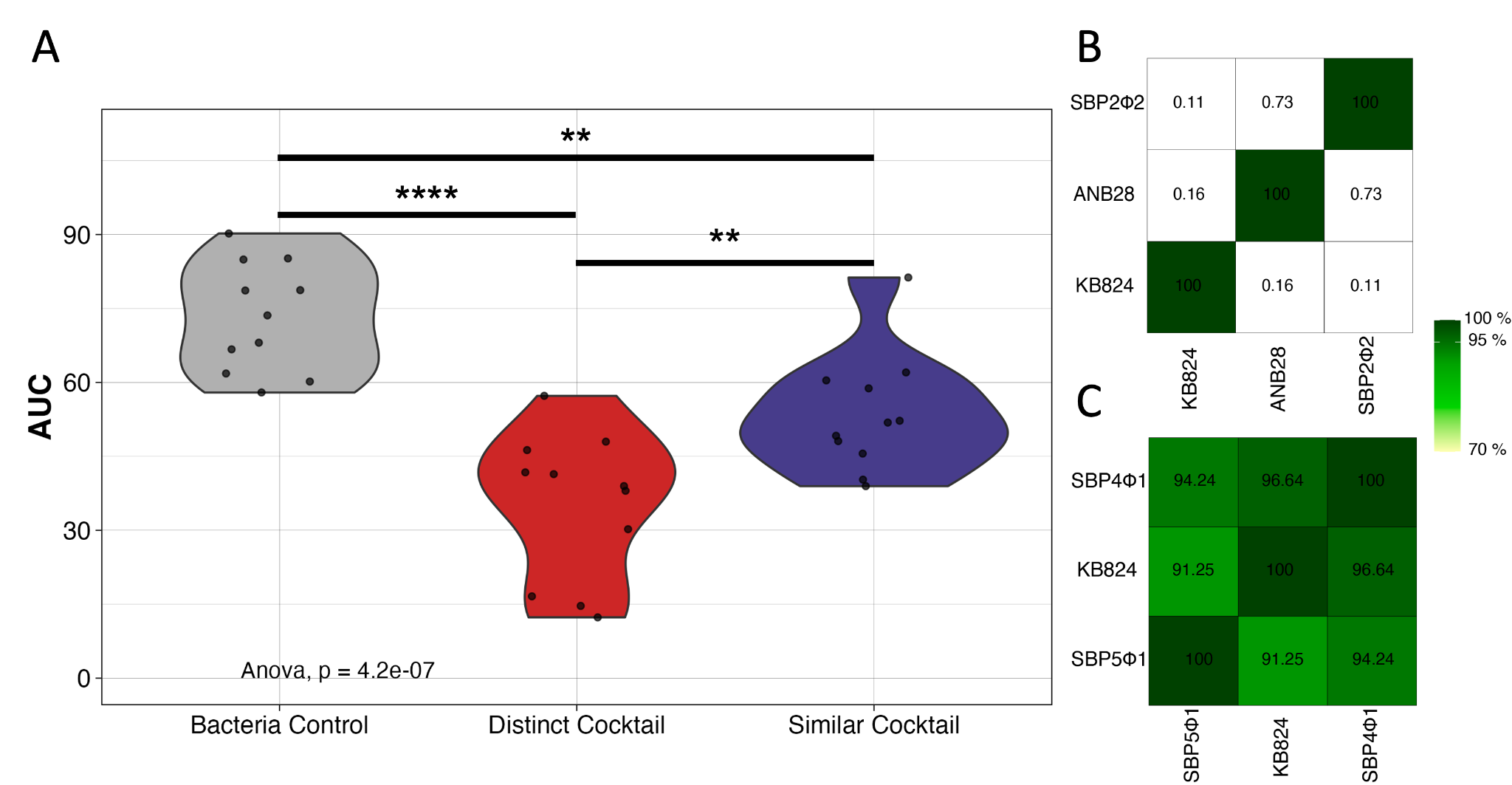
**

**Supplemental Figure S5. Exploring genetically diverse phage cocktail designs on *S. maltophilia* Strains.** (**A**) Log-phase strains were exposed to two different three-phage cocktails (MOI 1; based on host bacteria B28B). An area under the curve (AUC) analysis of 40-hour growth curve data (at 37°C) was conducted, followed by a one-way ANOVA. The main effect of the cocktail design was statistically significant and large (F(2, 30) = 24.90, p < .001; Eta² = 0.62, 95% CI [0.42, 1.00]). Tukey’s HSD Test for multiple comparisons revealed that all conditions were statistically different, with the bacterial control and distinct cocktail conditions showing the largest difference. Violin plots of AUC are shown, and each data point represents a different STM strain. Pairwise intergenomic distances/similarities of viral genomes were analyzed using Virus Intergenomic Distance Calculator (VIRIDIC) (68). (**B**) The distinct phage cocktail (ANB28, KB824, and SBP2Φ2) and (**C**) The similar phage cocktail (KB824, SBP4Φ1, and SBP5Φ1). Data visualization was done with R and ggplot2 (74, 85). *Significant levels*: p<0.01 (**), and p < 0.0001(****).

**
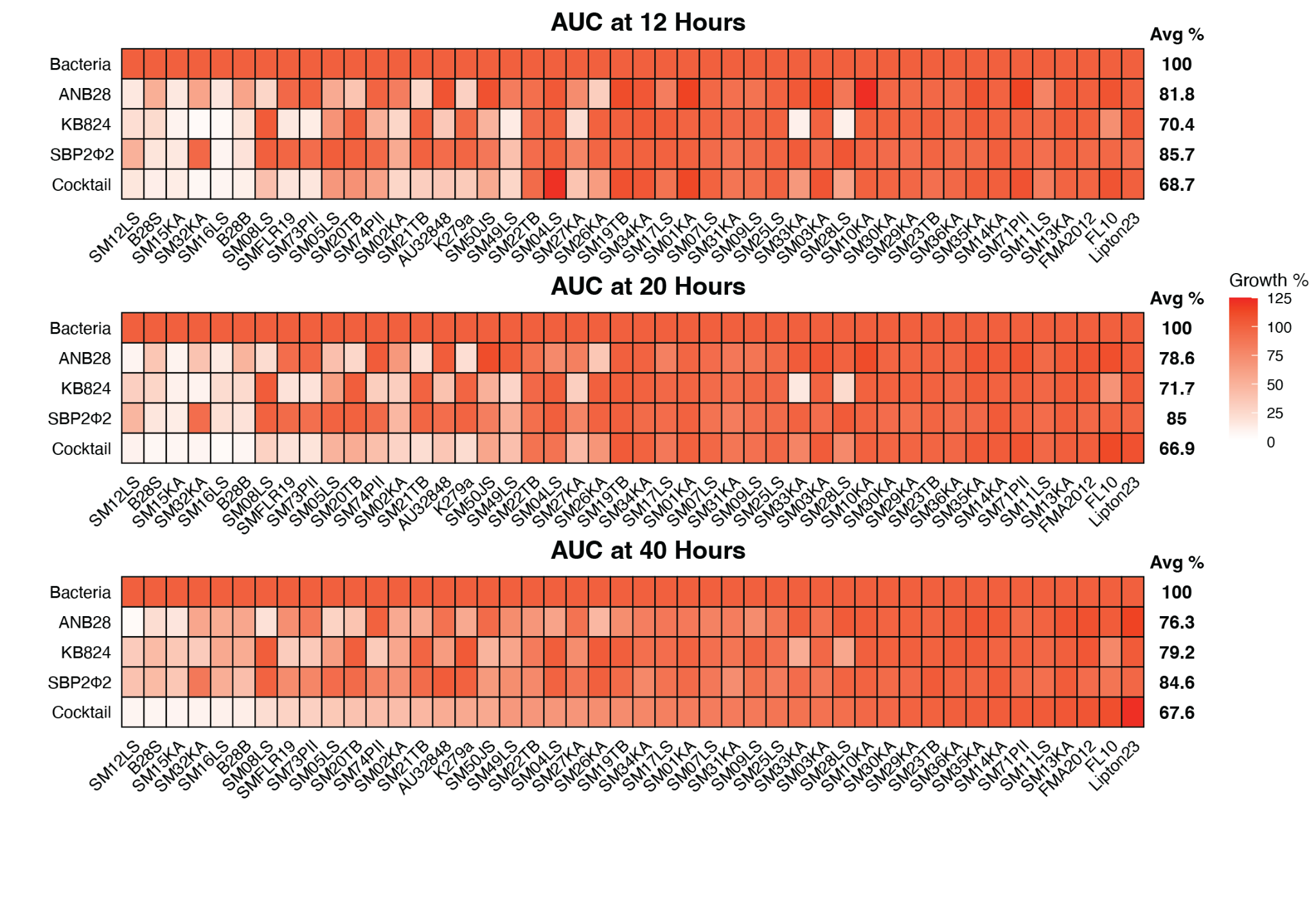
**

**Supplemental Figure S6. Host range of the three-phage cocktail against a cohort of clinically relevant *S. maltophilia* strains.** Strains were grown to a log phase (OD600 0.1) and exposed to Phage ANB28, KB824, SBP2Φ2, and a three-phage cocktail comprising all three phages in a 96-well plate setup. Phages were exposed at MOI 1 based on the titer of host strain B28B. Growth curve data (OD600) was collected from the Log Phase600 plate reader for 48 hours at 37°C. The area under the curve (AUC) was calculated for 12, 20, and 40 hours using the r package “gcplyr” after blank adjustment (87). The growth percentage was calculated using the following equation: [(1-(AUC_control_ - AUC_phage_)/AUC_control_)*100]. Darker red indicates a growth condition similar to bacteria-only control, while lighter shade indicates lysis (or reduced bacterial growth). Averages across each condition were calculated and listed on the right of the heatmap. Data is represented by three technical replicates.
